# Antioxidant and Immunomodulatory Properties of Chia Protein Hydrolysates in Primary Human Monocyte–Macrophage Plasticity

**DOI:** 10.3390/foods11050623

**Published:** 2022-02-22

**Authors:** Alvaro Villanueva-Lazo, Sergio Montserrat-de la Paz, Elena Grao-Cruces, Justo Pedroche, Rocio Toscano, Francisco Millan, Maria C. Millan-Linares

**Affiliations:** 1 Plant Protein Group, Department of Food and Health, Instituto de la Grasa-CSIC, Carretera de Utrera Km 1, Campus Universitario Pablo de Olavide, Edificio 46, 41013 Seville, Spain; alvarovillanueva@ig.csic.es (A.V.-L.); j.pedroche@csic.es (J.P.); fmillanr@ig.csic.es (F.M.); 2 Department of Medical Biochemistry, Molecular Biology and Immunology, School of Medicine, Universidad de Sevilla, Av. Dr. Fedriani 3, 41071 Seville, Spain; egrao@us.es (E.G.-C.); mtoscanos@us.es (R.T.); mmlinares@us.es (M.C.M.-L.)

**Keywords:** chia, *Salvia hispanica* L., immunonutrition, myeloid cells, oligopeptides, food-derived peptides

## Abstract

Chia (*Salvia hispanica* L.) seed has high potential in the development of functional food due to its protein content with a special amino acid profile. Among the hematopoietic-derived cells, monocytes are endowed with high plasticity, responsible for their pro- and anti-inflammatory function in M1 and M2 phenotype polarization, respectively. Indeed, monocytes are involved in several oxidative- and inflammatory-associated disorders such as cancer, obesity, and cardiovascular and neurodegenerative diseases. This study was designed to investigate the role of chia protein hydrolysates (CPHs) in primary human monocyte–macrophage plasticity response using biochemical, RT-qPCR, and ELISA assays. Our results showed that CPHs reduce ROS and nitrite output, as pro-inflammatory cytokine secretion, and enhance the expression and release of anti-inflammatory cytokines. In addition, CPHs reverse LPS-associated M1 polarization into M2. These findings open new opportunities for developing nutritional strategies with chia as a dietary source of biopeptides to prevent the development and progression of oxidative- and inflammatory-related diseases.

## 1. Introduction

Healthy dietary habits are not enough to treat disease status, and it is necessary to find novel drug treatments and to develop new functional components, which can act as preventive therapy of several diseases related to oxidative and inflammatory chronic states [1]. Hence, food-derived functional or bioactive compounds such as peptides can be included in the development of functional foods. Bioactive peptides might be obtained by gastrointestinal digestion, fermentation, or controlled hydrolysis processes using exogenous proteases [2]. Small peptides and amino acids might cross the natural intestinal barrier, enter the bloodstream, and play an important role in the immune system controlling oxidative and inflammatory pathways [3]. Regarding extensive hydrolysates of exogenous proteases, in vitro studies with seed protein hydrolysates have shown antioxidant biological activity, cholesterol lowering capacity, immunomodulatory properties, and angiotensin-converting enzyme (ACE) inhibition, among others [4]. For instance, the literature reports many legume hydrolysates which exert biological activity [5], such as anti-inflammatory and immunomodulatory effects of soy [6] and in the GPETAFLR peptide (Gly-Pro-Glu-Thr-Ala-Phe-Leu-Arg), isolated from lupine hydrolysates [7]. In addition, biological activity has been found in other vegetable seeds such as rice bran hydrolysates, promoting arteriosclerosis resolution [8], and hemp hydrolysates, preventing oxidation and neuroinflammation [9].

Chia (*Salvia hispanica* L.) is a native plant from the central region of South America [10] and is considered as a “superfood” due to its functional properties [11]. Chia has a high content of beneficial fatty acids as linolenic acid (C18:3, ω−3), linoleic acid (C18:2, ω−6), and oleic acid (C18, ω−9), which are related to the reduction in cholesterol levels and blood pressure [12]. Chia seeds are also rich in antioxidants, B vitamins, minerals, and fiber, their protein content (15–24%) has been highlighted in comparison to other seeds [13,14,15,16,17]. The sulfur-containing amino acid content as well as arginine, aspartic acid, and glutamic acid content exert important functions in protein functionality [16,18,19,20,21]. Therefore, chia gives the opportunity to obtain bioactive peptides, which exert beneficial effects on human health. Chia hydrolysates have been demonstrated to be a very efficient functional ingredient in a wide range of foods, as ACE inhibitors [22], and as anti-inflammatory agents [23]. Other recent studies have reported that peptides from the enzymatic hydrolysis of chia protein have antibacterial activity against Gram-positive (*S. aureus*) and Gram-negative (*E. coli*) microorganisms and inhibit cholesterol synthesis [24]. However, to our knowledge, the anti-oxidant and anti-inflammatory effects of chia on monocyte polarization and plasticity have not been described yet.

In recent years, oxidative stress and inflammation have been linked to immune cells and chronic non-communicable diseases such as cancer, cardiovascular disease, Alzheimer’s, Parkinson’s, arthritis, diabetes, and obesity, which are responsible for at least 70% of mortality around the world [25]. Particularly, human primary monocytes have been widely used in studies of chronic inflammatory status [7,9,26,27]. Through their plastic nature, monocytes can exert multiple roles during the immune response course [28]. Normally, monocytes are differentiated in a simple manner into two subsets: classical monocytes in M1 polarization status, which exert pro-oxidant and pro-inflammatory response, and non-classical monocytes in M2 polarization status, which exert ant-oxidative and anti-inflammatory response. The M1 and M2 polarization status may be differentiated by the expression of CD14 and CD16 surface receptors [29,30,31]. Hence, macrophage activation can occur through the recognition of pathogen-associated molecular pattern (PAMP) pathway between lipopolysaccharide (LPS) molecules and pathogen recognition receptors, including toll-like receptors (TLRs), proteins that play a key role in the innate immune system, with nuclear factor-kappa B (NF-κb) activation [32]. In this connection, interferon gamma (IFNγ) is one of the most potent inducers of classical macrophage activation (M1 macrophages) and is responsible for oxidant and pro-inflammatory agents [33]. In addition, IL-4 is one of the most potent inducers of non-classical macrophage activation (M2 macrophages), and the production of anti-inflammatory cytokines establishes the wound-healing state that accompanies the resolution of the inflammatory state and the catabasis of adaptive immune responses [34].

Within the framework of these criteria, the main objective of this research was to study the antioxidant and anti-inflammatory effects of chia (*Salvia hispanica* L.) protein hydrolysate on primary human monocytes, as well as its ability to re-program the monocyte–macrophage system.

## 2. Materials and Methods

### 2.1. Isolation of Chia Protein

Chia seeds (*Salvia hispanica* L.) were supplied by the Autonomous University of Nuevo Leon (Saint Nicholas de Los Garza, Mexico). Chia protein isolate (CPI) was obtained by Plan Protein Group’s pilot plant in the Instituto de la Grasa (IG-CSIC, Seville, Spain) from defatted chia flour using the method of Lqari [35] with some modifications. Briefly, defatted chia meal was extracted with 0.25% Na_2_SO_3_ (*w*/*v*) at pH 10.5 for 1 h. After centrifuging the extract in a decanter for 15 min, supernatant was recovered, and pellet was extracted again. Both supernatants were adjusted to the isoelectric point of chia protein (pH 4.0) and centrifuged again. The resulting precipitate was washed with distilled water, adjusted to pH 4.0, and centrifuged to remove residual salts and other non-protein compounds. Finally, the precipitated proteins were atomized and stored at room temperature.

### 2.2. Production of Chia Protein Hydrolysate

According to Villanueva-Lazo et al. [36], chia protein hydrolysate (CPH) was obtained from CPI after 15 min of hydrolysis with Alcalase 2.4 L (Novozymes, Madrid, Spain), since it was the hydrolysate that showed better antihypertensive and antioxidant properties. Briefly, 50 g of CPI was dissolved in water in a ratio of 7.5% (*w*/*v*). After adjusting pH to 8.0, hydrolysis was carried out with commercial enzyme Alcalase 2.4 L (Novozymes, Madrid, Spain) at 50 °C and with an enzyme concentration of 0.3 Anson units (AU)/g protein, maintaining pH at 8 with 1 N NaOH. Incubation with this enzyme was carried out for 15 min. Next, the enzyme was inactivated at 90 °C for 10 min by immersing the sample in a thermostatic bath. The degree of hydrolysis reached, defined as the percentage of peptide bonds cleaved, was calculated by determining protein content and the number of free amino groups with the TNBS method [37]. The sample was hydrolyzed with 6 N HCl for 24 h to determine the total number of amino groups.

### 2.3. Chemical Characterization of Chia Isolate and Protein Hydrolysate

The protein content determination was carried out by elemental microanalysis of nitrogen content (x6.25) using a LECO CN-828 analyzer (St. Joseph, MI, USA). The amino acid determination was carried out using the method of Alaiz et al. with slight modifications [38]. The samples (4–6 mg) were hydrolyzed with 4 mL of HCl 6N at 110 °C for 24 h in tubes sealed under nitrogen. The acid hydrolysate was derivatized with diethyl ethoxymethylenemalonate, and D,L-α-aminobutyric acid was used as the internal standard. Amino acid content was determined with ultra-high-performance liquid chromatography, using a binary system gradient, (A) 25 mM sodium acetate and 0.02% sodium azide (pH 6.0) and (B) acetonitrile, in an Acquity Arc equipped with a 2998PDA Detector, a Sample Manager FTN-R, and a Quaternary Solvent Manager-R (Acquity Arc, Waters Corporation, Milford, MA, USA) and a 3 × 150 mm reversed-phase column (XSelect^®^ HSS T3, 2.5 µm) (Waters Corporation, Milford, MA, USA). The Yust method was used to determine the tryptophan content [39]. Samples (20–24 mg) were hydrolyzed with 3 mL of 4 N NaOH at 110 °C for 4 h in closed tubes and under a nitrogen atmosphere. Moisture was calculated by weight difference between initial sample and dry sample at 110 °C to constant weight. Ash content was also determined by gravimetry, with samples being incinerated at 550 °C for 36 h using the direct ignition method. Fiber content was determined according to Lee [40]. This method is based on digestion of samples by thermostable α-amylase enzymes, protease, and amyloglucosidase and subsequent determination of resulting residue by gravimetry.

### 2.4. Isolation of Primary Human Monocytes

Peripheral blood mononuclear cells (PBMCs) were obtained from buffy coats donated by Centro Regional de Transfusiones Sanguíneas y Banco de Tejidos de la provincia de Sevilla y Huelva. PBMCs were isolated by centrifugation on a gradient with Ficoll (Sigma, Madrid, Spain) [31] and monocytes were separated from PBMCs by positive selection using CD14 microbeads and LS columns on a midiMACS system (Miltenyi Biotec, Madrid, Spain) according to the manufacturer’s instructions. After isolation, monocytes were suspended in RPMI 1640 medium supplemented with L-glutamine, penicillin, streptomycin, and 1% heat-inactivated fetal bovine serum. For treatments, 5 × 10^5^ monocytes/well were seeded in 24-well plates in presence or absence of LPS (100 ng/mL) (Sigma, Madrid, Spain) and treated with CPH at 50 and 100 μg/mL for 24 h.

### 2.5. Cell Viability (MTT)

Cell viability was determined using the 3-(4,5-dimethyltiazol-2-yl)-2,5-diphenyltetrazolium bromide (MTT) method. Monocytes were seeded in a 96-well plate in a 1 × 10^5^ density and incubated (5% CO_2_ at 37 °C in a CO_2_ incubator) (Thermo Con Electron Corporation, Waltham, MA, USA) for 24 h in the presence of CPH at the concentration range 25–200 μg/mL. Then, an aqueous solution of MTT was added at 0.5 mg/mL, and the plate was incubated for 2 h (5% CO_2_ at 37 °C in a CO_2_ incubator). Formazan crystals were dissolved in dimethyl sulfoxide (DMSO), and the absorbance was determined by using a Multiskan Spectrum (Thermo Labsystems, Gulph Mills, PA, USA) at 570 nm with 620 nm correction.

### 2.6. Differentiation and Polarization to Macrophages M1/M2

Monocytes (5 × 10^5^ per well) were incubated for 6 days in the presence of recombinant human M-CSF (25 ng/mL) to obtain differentiated M0 macrophages. These cells were then cultured in RPMI 1640 supplemented with L-glutamine, penicillin, streptomycin, and 10% FBS. For polarization to M1 and M2, M0 macrophages were exposed to LPS (100 ng/mL) plus IFNγ (20 ng/mL) and IL-4 (20 ng/mL), respectively, for an additional 24 h. To evaluate the effect of CPH on macrophage polarization, M1 macrophages were exposed to 50 and 100 μg/mL of CPH for 24 h.

### 2.7. Reactive Oxygen Species (ROS) Production

ROS intracellular levels were determined using CellROX reagent (Thermo Fisher Scientific, Madrid, Spain). After in vitro stimulation with LPS at 100 ng/mL, monocytes were exposed to 50 and 100 μg/mL of CPH for 24 h and then with CellROX (5 μM) for 30 min. Cells were washed with phosphate-buffered saline (PBS), and the fluorescence signal was analyzed in a Fluoroskan Microplate Fluorometer (Thermo Fisher Scientific). Cell autofluorescence was measured under the same conditions, but without adding CellROX. Data shown refer to percentage of intracellular ROS production and to comparison with a positive control (100% ROS production) after cell treatment in LPS presence.

### 2.8. Nitrite Production

The nitric oxide (NO) production was measured in co-cultured supernatant using the Griess method in previously LPS-activated monocytes incubated with CPH at 50–100 μg/mL. Griess reagent (Sigma-Aldrich, Madrid, Spain) was added at the same volume of co-culture supernatant in a 96-well plate. The absorbance was measured in triplicate samples at 540 nm using the Multiskan Spectrum. The NO concentration was calculated from the calibration curve from serial dilution of NO_2_.

### 2.9. Cytokine Quantification

Levels of tumoral necrosis factor (TNF)-α, interleukin (IL)-1β, IL-6, and IL-10 in culture supernatants were determined by an enzyme-linked immunosorbent assay (ELISA), following the instructions of the manufacturer (Diaclone, Besancon, France).

### 2.10. RNA Isolation and RT-qPCR

Total RNA was extracted using Trisure Reagent (Bioline). An A_260_/A_280_ ratio in a NanoDrop ND-1000 spectrophotometer (Thermo Scientific, Madrid, Spain) was used to assay RNA quality. Subsequently, RNA (1 µg) was subjected to reverse transcription (iScript, Bio-Rad, Madrid, Spain). An amount of 10 ng of resulting cDNA was used as a template for real-time PCR amplifications. The mRNA levels of specific genes were determined in a CFX96 system (Bio-Rad) containing primer pairs for either gene or for glyceraldehyde 3-phosphate dehydrogenase (*GAPDH*) and hypoxanthine phosphoribosyltransferase 1 (*HPRT*) as housekeeping genes. All amplification reactions were performed in triplicate with average threshold cycle (Ct) numbers of magnitude of change in mRNA expression of candidate genes, quantified with the standard 2^−(ΔΔCt)^ method. All data were normalized to endogenous reference (*GAPDH* and *HPRT*) gene content and expressed as percentage of controls. Primers used for the amplifications are detailed in Table 1.

### 2.11. Statistical Analysis

All values are expressed as arithmetic means ± standard deviation (SD). Data were evaluated with GraphPad Prism Version 6.01 software (San Diego, CA, USA). The statistical significance of any difference in each parameter among the groups was evaluated by one-way analysis of variance (ANOVA), following Tukey’s multiple comparison test as a post hoc test. *p* values less than 0.05 were considered statistically significant.

## 3. Results

### 3.1. Chemical Characterization of the CPI and CPH

CPH was obtained from CPI after 15 min hydrolysis with Alcalase 2.4 L, reaching a degree of hydrolysis of 36.2% and a protein content of 75.03%. Table 2 shows protein, moisture, ash, fiber, and other contents presented in CPH.

The amino acid profile of CPH is shown in Table 3. In addition, CPI amino acid analysis results and FAO/WHO/UNU nutritional recommendations for adults for essential amino acids are presented. It highlights the presence of negatively charged amino acids, glutamic acid and aspartic acid (176.2 and 85.2 mg/g protein, respectively), as well as arginine (108 mg/g protein) in CPI. These results are similar to those obtained by CPH.

### 3.2. CPH Decreases Oxidative Stress in Human Primary Monocytes

**Figure 1:**
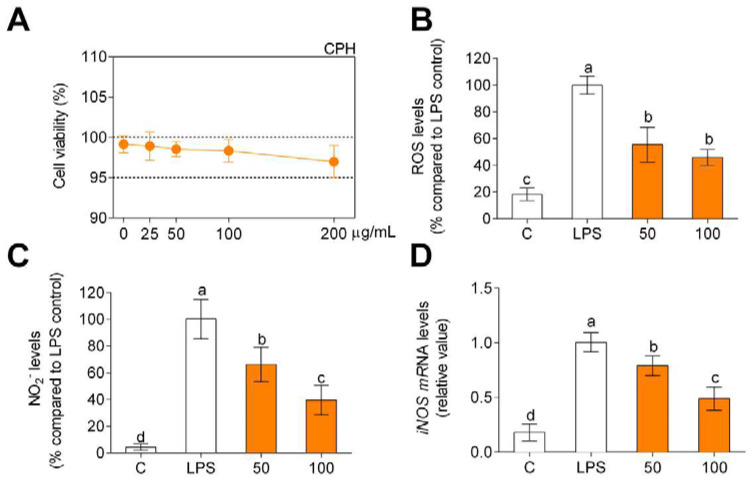
Cell viability (**A**), production of ROS (**B**), and nitriles (**C**) expressed as fluorescence/absorbance and iNOS mRNA levels (**D**) compared to LPS-stimulated cells after CPH treatment at 50 and 100 μg/mL for 24 h in human primary monocytes. The values are presented as means ± SD (*n* = 3). Different letters denote statistical differences (*p* < 0.05).

Once the hydrolysate was characterized, the anti-inflammatory and antioxidant effect tests were carried out, and the first step was to verify if the hydrolysate produces a cytotoxic effect on the primary human monocytes. For this purpose, they were incubated for 24 h up to doses of 200 µg/mL, verifying that there was no cytotoxic effect (Figure 1A). Taking into account the MTT assay, CPH doses of 50 and 100 µg/mL were used to test antioxidant and anti-inflammatory activity of CPH. LPS significantly increased both intracellular ROS (Figure 1B) and nitrite (Figure 1C) compared to unstimulated cells (C). On the other hand, as can be seen in Figure 1B, the cells that were stimulated with LPS and treated with CPH decreased ROS production, a decrease that is greater, although not statistically significant, when increasing the concentration of the CPH. The same effect is observed in Figure 1C regarding the production of nitrites. In this case the increase in the concentration of CPH produces a decrease in the production of nitrites which is statistically significant. In addition, LPS-stimulated cells showed higher iNOS gene expression compared to unstimulated cells (Figure 1D), and the CPH treatment decreased iNOS gene expression in LPS-stimulated human primary monocytes. This decrease was significantly less when increasing the concentration of CPH.

### 3.3. CPH Decreases Pro-Inflammatory Cytokine Levels and Gene Expression in Human Primary Monocytes

**Figure 2:**
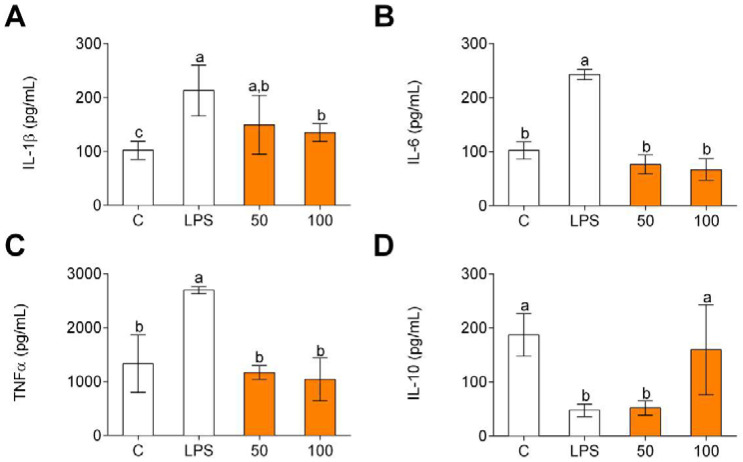
Pro-inflammatory (**A**–**C**) and anti-inflammatory (**D**) cytokine production in human primary LPS-stimulated monocytes after CPH treatment at 50 and 100 μg/mL for 24 h. The values are presented as means ± SD (*n* = 3). Different letters denote statistical differences (*p* < 0.05).

Pro-inflammatory cytokine (IL-1β, IL-6, and TNFα) and anti-inflammatory cytokine (IL-10) gene expression was evaluated in order to determine potential anti-inflammatory effects of CPH. LPS significantly increased the production of IL-1β (Figure 2A), IL-6 (Figure 2B), and TNFα (Figure 2C) compared to unstimulated cells (C). The pro-inflammatory gene expression increase was significantly reduced by CPH at 100 μg/mL. Conversely, LPS decreased the production of anti-inflammatory cytokine IL-10 (Figure 2D), and CPH at 100 µg/mL restored the production of this cytokine to unstimulated cell levels. 

**Figure 3:**
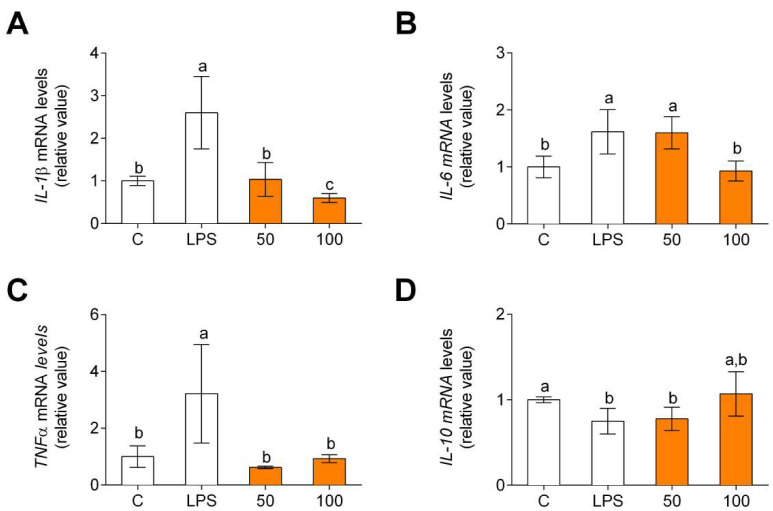
Pro-inflammatory (**A**–**C**) and anti-inflammatory (**D**) cytokine gene expression in human primary LPS-stimulated monocytes after CPH treatment at 50 and 100 μg/mL for 24 h. The values are presented as means ± SD (*n* = 3). Different letters denote statistical differences (*p* < 0.05).

In line with the results obtained for cytokine gene expression, LPS increased the release of pro-inflammatory cytokines (Figure 3A–C) and decreased the release of IL-10 (Figure 3D). In all these cases, CPH significantly decreased the release of pro-inflammatory cytokines and increased IL-10 level.

### 3.4. CPH Promotes M2 Polarization and Decreases the Pro-Inflammatory State in Human Monocyte-Derived Macrophages

**Figure 4:**
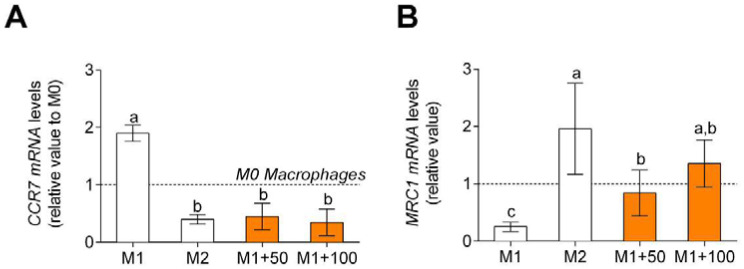
Macrophage polarization marker gene expression, CCR7, M1 polarization marker (**A**) and MRC1, M2 polarization marker (**B**). M0 macrophages were incubated with LPS + IFNγ (M1 control), IL-4 (M2 control), or LPS + IFNγ + CPH (50 and 100 μg/mL for 24 h). Values are presented as means ± SD (*n* = 3). Different letters denote statistical differences (*p* < 0.05).

Different microenvironments and signals promote macrophage polarization. Macrophages show specific phenotypes that correspond to classical (M1) and alternative (M2) phenotypes. Non-polarized macrophages, after LPS and IFNγ incubation, were differentiated into M1 macrophages. Macrophages showed an increase in CCR7 gene expression, M1 polarization marker (Figure 4A), and a decrease in MRC1 gene expression. An M2 polarization marker (Figure 4B) was found. When M1 polarized macrophages were treated with CPH, CCR7 gene expression decreased, and MRC1 gene expression increased.

**Figure 5:**
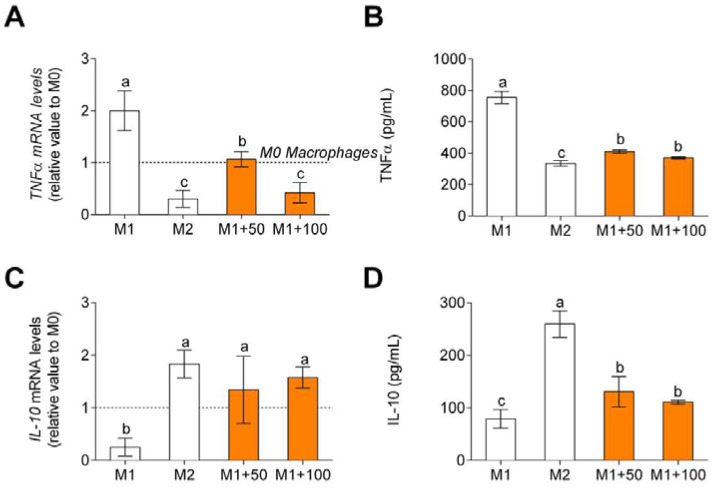
TNFα, M1 macrophage polarization-associated cytokine, gene expression (**A**), and release (**B**) and IL-10, M2 macrophage polarization-associated cytokine, gene expression (**C**), and release (**D**). M0 macrophages were incubated with LPS + IFNγ (M1 control), IL-4 (M2 control), or LPS + INFγ + CPH (50 and 100 μg/mL for 24 h). Values are presented as means ± SD (*n* = 3). Different letters denote statistical differences (*p* < 0.05).

Non-polarized macrophages after LPS and IFNγ treatment showed an increase in TNFα, pro-inflammatory cytokine, gene expression and release (Figure 5A), and release (Figure 5B) and a decrease in IL-10, anti-inflammatory cytokine, gene expression (Figure 5C), and release (Figure 5D). When treated with CPH, pro-inflammatory cytokine gene expression and release decreased, and anti-inflammatory cytokine gene expression and release increased.

## 4. Discussion

Although in recent years chia seeds (*Salvia hispanica* L.) have become increasingly important for the food industry due to their high nutritional content and beneficial health effects that arise from their consumption [2], there is limited research on the benefits of their protein hydrolysates [13,19].

Enzymatic hydrolysis of protein-rich by-products from the agri-food industry can lead to protein hydrolysates with better functional properties and biological activities to be used as ingredients in preparation of functional foods or to elaborate new nutraceuticals. In this work, we obtained CPH by hydrolysis with Alcalase 2.4 L for 15 min from the CPI with high protein and essential amino acids content, with the added value of high solubility [36], characteristic of protein hydrolysates. 

Once CPH was obtained, the in vitro functionality in oxidative and inflammatory state in human primary monocytes was studied. Monocytes are essential cells in the development and perpetuation of inflammatory state, and they exhibit different phenotypes according to the expression of surface markers CD14 and CD16 [27]. The main phenotypes of circulating monocytes belong to CD14^+^, CD16^+^, and CD14^+^ phenotype CD16^++^ [26]. In response to an inflammatory stimulus, such as LPS, classical monocytes accumulate in the first instance at sites of inflammation [7,9]. These monocytes are highly invasive and can differentiate into pro-inflammatory M1 macrophages, acting during the acute phase of inflammation. In a stable state, non-classical monocytes are found at the vascular endothelium and become resident macrophages upon infiltrating inflamed tissue. During the late phase of inflammation, non-classical differentiated monocytes (formally M2 macrophages) acquire an anti-inflammatory phenotype, which contributes to the arrest of activated leukocytes and the repair of damaged tissues [29,30]. 

In our study, primary human monocytes isolated from buffy coat and stimulated with LPS showed an increase in oxidative stress, increasing ROS and NO production, such as M1 macrophage polarization. CPH decreased oxidative stress in human primary monocytes, specifically reducing ROS and NO production. Our observations suggest that the inhibition of iNOS expression, which is induced by LPS, could be one of the CPH mechanisms to decrease oxidative stress and inflammation in primary human monocytes. CPH effects on oxidative stress are clinically relevant, since chronic oxidative stress can accelerate and continuously stimulate macrophage infiltration in those damaged tissues, and macrophages can develop an immune reaction. Chronic inflammation enhances the development of inflammatory diseases [23].

In line with previous results, stimulation with LPS produced an increase in the release and gene expression of pro-inflammatory cytokines IL-1β, IL-6, and TNFα and a decrease in IL-10, an anti-inflammatory cytokine. This balance of cytokines in human primary monocytes is related to the onset and development of different diseases, including cardiovascular diseases, because it enhances the development of atherosclerotic plaque [23] and the chronic inflammation course with pro-inflammatory cytokine release in conditions such as obesity and fatty liver disease [41,42]. CPH could be tested in different tissues to corroborate the anti-inflammatory effects of the protein hydrolysate. CPH reversed pro-inflammatory state induced by LPS in human primary monocytes, probably acting at transcription factor NF-κB level, although molecular mechanisms by which CPH acts should be studied in future trials.

After studying the effect of CPH on human monocytes, we studied its effects on macrophage polarization. Pro-inflammatory macrophages polarize to M1 phenotype under the influence of LPS and/or INFγ and to M2 with exposure to IL-4. A possible strategy to control chronic inflammation is modulation of macrophage plasticity. It should be noted that CPH polarized macrophages towards M2 anti-inflammatory phenotype, increasing MRC1 and IL-10 gene expression and anti-inflammatory cytokine release. In addition, gene expression of CCR7 and TNFα, characteristic markers of the pro-inflammatory M1 phenotype, was reduced. The regulation of these markers and cytokines derived from polarized macrophages plays a key role in a variety of human inflammatory diseases [28,30,31].

CPH has shown clinically relevant anti-inflammatory functions in monocytes, reducing their oxidative and inflammatory status. Monocytes are crucial cells to immune response, as monocyte inflammatory and oxidative status influences the immune response. An imbalance in monocyte oxidative and inflammatory status could compromise the reparative reaction of the immune response (essential for the normal tissue recovery), leading to chronic inflammation, which is the onset of numerous diseases. Hence, CPH actions affecting monocyte oxidative and inflammatory status could reduce chronic inflammation.

## 5. Conclusions

Our study complements previous findings by demonstrating that chia, specifically its CPH, strongly promotes polarization of human primary monocytes and mature macrophages towards a less inflammatory phenotype; therefore, it is tempting to suggest that ingestion of chia and its protein hydrolysate may intervene in re-programming of monocytes and activation of macrophages in inflamed human tissues. CPH presents an ideal initial framework to continue the study of its functionality in preclinical models which study the prevention and treatment of inflammation-related conditions and evaluate its possible incorporation as a dietary supplement in food matrices as a natural bioactive compound.

## Figures and Tables

**Table 1:** Primer sequences for RT-qPCR gene expression analysis.

Target	GenBank Accession Number	Forward Reverse	Sequence (5′→3′)
*iNOS*	NM_ 000625	Forward Reverse	ACCCAGACTTACCCCTTTGG GCCTGGGGTCTAGGAGAGAC
*IL1β*	NM_000576	Forward Reverse	GGGCCTCAAGGAAAAGAATC TTCTGCTTGAGAGGTGCTGA
*IL6*	NM_000600	Forward Reverse	TACCCCCAGGAGAAGATTCC TTTTCTGCCAGTGCCTCTTT
*TNFα*	NM_000594	Forward Reverse	TCCTTCAGACACCCTCAACC AGGCCCCAGTTTGAATTCTT
*IL10*	NM_000572	Forward Reverse	GCCTAACATGCTTCGAGATC TGATGTCTGGGTCTTGGTTC
*CCR7*	NM_007719.2	Forward Reverse	GTGTGCTTCTGCCAAGATGA CCACGAAGCAGATGACAGAA
*MRC1*	NM_ 138806	Forward Reverse	GGCGGTGACCTCACAAGTAT ACGAAGCCATTTGGTAAACG
*HPRT*	NM_001289746	Forward Reverse	ACCCCACGAAGTGTTGGATA AAGCAGATGGCCACAGAACT
*GAPDH*	NM_001289746	Forward Reverse	CACATGGCCTCCAAGGAGTAAG CCAGCAGTGAGGGTCTCTCT

**Table 2:** Chemical composition (g/100 g) of chia protein products. CPI, chia protein isolate; CPH, chia protein hydrolysate obtained after 15 min of hydrolysis with Alcalase 2.4 L.

	CPI	CPH
Protein (%)	82.85 ± 0.11	75.03 ± 0.45
Moisture (%)	4.80 ± 0.30	7.18 ± 0.08
Ash (%)	0.13 ± 0.09	6.45 ± 0.17
Fiber (%)	11.01 ± 0.80	11.20 ± 0.70
Other * (%)	1.21	0.14

Data are means ± SD, *n* = 3. * 100 − (Protein(%) + Moisture (%) + Ash(%) + Fiber(%)).

**Table 3:** Summary of the adult indispensable amino acid composition and requirements of chia protein products. CPI, chia protein isolate; CPH, chia protein hydrolysate obtained after 15 min of hydrolysis with Alcalase.

Indispensable Amino Acid Protein	CPI	CPH	2007 FAO/WHO/UNU ^a,b^
Histidine	37.9 ± 2.9	39.2 ± 0.5	15
Isoleucine	35.2 ± 0.3	35.1 ± 0.0	30
Leucine	70.4 ± 4.6	72.2 ± 0.7	59
Lysine	46.4 ± 1.4	48.1 ± 0.6	45
Methionine + cysteine	40.5 ± 1.9	40.3 ± 1.2	22
Methionine	21.9 ± 1.8	21.1 ± 2.1	16
Cysteine	18.6 ± 1.9	19.2 ± 0.4	6
Phenylalanine + tyrosine	136.7 ± 1.9	135.5 ± 1.1	38
Threonine	38.4 ± 2.1	38.0 ± 0.3	23
Tryptophan	48.1 ± 0.6	49.3 ± 0.3	6
Valine	46.9 ± 0.9	47.6 ± 0.8	39
Total indispensable amino acids	500.5	505.3	277
**Dispensable Amino Acid Protein**			
Aspartic acid	85.2 ± 1.7	84.5 ± 1.3	
Glutamic acid	176.2 ± 2.7	178.3 ± 1.9	
Serine	71.9 ± 1.6	70.2 ± 0.7	
Glycine	44.5 ± 2.4	45.0± 0.6	
Arginine	108.0 ± 2.9	105.3 ± 1.1	
Alanine	48.0 ± 1.2	49.1 ± 0.4	
Proline	23.1± 1.0	17.6 ± 0.3	
Total dispensable amino acids	556.9	550	

Data are shown in % and ± SD, *n* = 3. ^a^ FAO/WHO/UNU. Scoring pattern mg/g protein requirement in adults. ^b^ FAO and FINUT 2017. Dietary protein quality evaluation in human nutrition. FAO Food and Nutrition Paper NO. 92.

## Data Availability

Data are contained within this article.
